# Possible linkages between the inner and outer cellular states of human induced pluripotent stem cells

**DOI:** 10.1186/1752-0509-5-S1-S17

**Published:** 2011-06-20

**Authors:** Shigeru Saito, Yasuko Onuma, Yuzuru Ito, Hiroaki Tateno, Masashi Toyoda, Akutsu Hidenori, Koichiro Nishino, Emi Chikazawa, Yoshihiro Fukawatase, Yoshitaka Miyagawa, Hajime Okita, Nobutaka Kiyokawa, Yohichi Shimma, Akihiro Umezawa, Jun Hirabayashi, Katsuhisa Horimoto, Makoto Asashima

**Affiliations:** 1 Computational Biology Research Center, National Institute of Advanced Industrial Science Technology (AIST), 2-4-7 Aomi, Koto-ku, Tokyo 135-0064, Japan; 2 INFOCOM CORPORATION, Sumitomo Fudosan Harajuku Building, 2-34-17, Jingumae, Shibuya-ku, Tokyo, 150-0001, Japan; 3 Research Center for Stem Cell Engineering, National Institute of Advanced Industrial Science Technology (AIST), Tsukuba Central 4, 1-1-1 Higashi, Tsukuba, Ibaraki 305-8562, Japan; 4 Research Center for Medical Glycoscience, National Institute of Advanced Industrial Science Technology (AIST), Tsukuba Central 2, 1-1-1 Umezono, Ibaraki 305-8568, Japan; 5 Department of Reproductive Biology, National Research Institute for Child Health and Development, 2-10-1 Ookura, Setagaya-ku, Tokyo 157-8535, Japan; 6 Department of Developmental Biology and Pathology, National Research Institute for Child Health and Development, 2-10-1 Okura, Setagaya-ku, Tokyo 157-8535, Japan; 7 Institute for Systems Biology, Shanghai University, Shangda Road 99, Shanghai 200444, China; 8 Department of Life Sciences (Biology), Graduate School of Arts and Sciences, The University of Tokyo, 3-8-1 Komaba, Meguro-ku, Tokyo 153-8902, Japan

## Abstract

**Background:**

Human iPS cells (hiPSCs) have attracted considerable attention for applications to drug screening and analyses of disease mechanisms, and even as next generation materials for regenerative medicine. Genetic reprogramming of human somatic cells to a pluripotent state was first achieved by the ectopic expression of four factors (Sox2, Oct4, Klf4 and c-Myc), using a retrovirus. Subsequently, this method was applied to various human cells, using different combinations of defined factors. However, the transcription factor-induced acquisition of replication competence and pluripotency raises the question as to how exogenous factors induce changes in the inner and outer cellular states.

**Results:**

We analyzed both the RNA profile, to reveal changes in gene expression, and the glycan profile, to identify changes in glycan structures, between 51 cell samples of four parental somatic cell (SC) lines from amniotic mesodermal, placental artery endothelial, and uterine endometrium sources, fetal lung fibroblast (MRC-5) cells, and nine hiPSC lines that were originally established. The analysis of this information by standard statistical techniques combined with a network approach, named network screening, detected significant expression differences between the iPSCs and the SCs. Subsequent network analysis of the gene expression and glycan signatures revealed that the glycan transfer network is associated with known epitopes for differentiation, e.g., the SSEA epitope family in the glycan biosynthesis pathway, based on the characteristic changes in the cellular surface states of the hiPSCs.

**Conclusions:**

The present study is the first to reveal the relationships between gene expression patterns and cell surface changes in hiPSCs, and reinforces the importance of the cell surface to identify established iPSCs from SCs. In addition, given the variability of iPSCs, which is related to the characteristics of the parental SCs, a glycosyltransferase expression assay might be established to define hiPSCs more precisely and thus facilitate their standardization, which are important steps towards the eventual therapeutic applications of hiPSCs.

## Background

Reprogramming of human and mouse fibroblasts to induced pluripotent stem cells (iPSCs) has been achieved by the expression of only four transcription factors, Oct4, Sox2, Klf4, and c-Myc, referred to as the “four factors” [1]. iPSCs hold great promise for human disease analyses and therapies, because they are highly similar to embryonic stem cells (ESCs) in their ability to self-renew and generate all three germ layers. A key question raised by transcription factor-induced reprogramming to self-renewal and pluripotency is how the four factors act to accomplish these changes in the inner and outer cell states.

The morphological changes accompanying the reprogramming of somatic cells to iPSCs can be visually identified by alkaline phosphatase staining. The changes in the outer cellular states are further monitored by characteristic molecular markers. In fact, the monoclonal antibodies currently used to define ESCs and iPSCs, including the globo-series glycosphingolipid epitopes SSEA-3 and SSEA-4, and the keratanase-sensitive glycoprotein associated epitopes Tra 1–60 and Tra 1–81, recognize glycan antigens [2-4]. Recently, global analyses of glycan signatures for pluripotency on the cell surface were reported, by direct observations of glycan structures by MALDI-TOF mass spectrometric and NMR spectrometric profiling in ESCs [5] and indirect observations of lectins by a lectin microarray in stem cells [6]. Furthermore, the extracellular matrix is also important for controlling cellular states through cell-cell interactions [7].

The inner cellular states also change during the remodeling of the somatic cell transcription and chromatin programs to the ES-like state, including the reactivation of the somatically silenced X chromosome, the demethylation of the Oct4 and Nanog promoter regions, and the genome-wide resetting of histone H3 lysine 4 and 27 trimethylation [8]. It is particularly important to determine whether the gene expression differences observed between hiPSCs and the corresponding parental cells actually reflect the differences between these pluripotent cell types, especially between hiPSCs and ESCs [9-12]. Gene expression signatures were reported for reprogrammed cell lines derived in different labs by various methods [13-15]. In addition, genome-wide mapping of transcription factor targets by ChIP, combined with microarrays or sequencing methods, can provide a foundation for understanding transcriptional networks [16-20]. Expanding the number of transcription factors analyzed by ChIP-based methods is especially informative in dissecting system level biological processes. In ESCs, some groups have used new methods for global target mapping to predict the target genes regulated by Oct4, Sox2, and Nanog, and these studies revealed the combinatorial occupancy of target gene promoters by these core factors, as well as both autoregulatory and feed-forward transcriptional circuits [16-20].

Here, we applied two methods, RNA profiling to uncover gene expression changes, and lectin profiling to survey glycan structure changes, to compare human iPSCs and parental somatic cells (SCs), including 51 cells of nine iPSC lines from four kinds of SCs, from amniotic mesodermal, placental artery endothelial, and uterine endometrium sources, and one available hiPSC line, MRC-5. The changes were computationally analyzed by a network approach [21] in conjunction with information on the gene binding from previous CHIP-seq studies and knowledge of the gene functions. The sum of these analyses uncovered novel expression, network, and lectin signatures that are unique to the hiPSC lines and differ from those of the parental cells. The following correspondence between the three signatures identified a few glycosyltransferases as novel candidates, due to the characteristic changes of their cellular surface states in hiPSCs, which shed light on a possible link between the inner and outer cellular states. Whether the hiPSC signatures described here actually play a functional role in bridging the gap between the two cellular states warrants extensive further investigations.

## Results and Discussion

### hiPSCs descended from different parent SCs are distinguishable by gene expression

To determine the gene expression signatures of hiPSCs, a detailed genome-wide expression analysis was performed to compare iPSCs and their parental SCs from amniotic mesodermal (AM), placental artery endothelial (PAE), uterine endometrium (UtE), and MRC-5 (MRC) cell sources (see additional file 1: Cell lines and numbers of passages analyzed in the present study, and Methods). In total, 51 cell samples of 13 cell lines (39 hiPSC samples of 9 hiPSC lines [22,23]) were analyzed in the present study, for a statistical comparison of the hiPSCs and the parental SCs (see additional file 2: Generation of iPSCs from human PAE cells).

**Figure 1:**
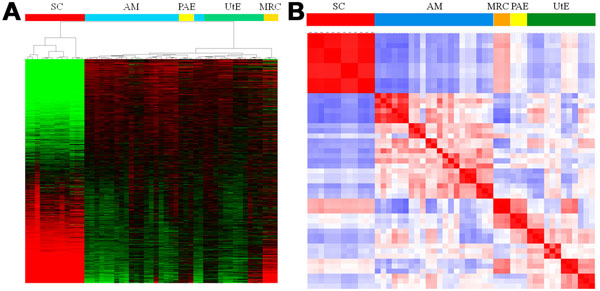
**Classification of hiPSCs and SCs from gene expression data** (*A*) Heat map and hierarchical clustering for all cells and genes. Cell types are indicated by colored bars, and the following abbreviations are used for the sources of the cell types of the human somatic cells (SCs) and induced pluripotent stem cells (hiPSCs): AM, amniotic membrane cell; PAE, placental artery cell; UtE, uterine endometrium cell; MRC, MRC-5 cell. Details are available in additional file 1. (*B*) Heat map of the correlation coefficient matrix for all of the cells. See also additional file 3.

Unsupervised hierarchical clustering of the gene expression data across the four hiPSC lines (AM, PAE, UtE, MRC) and their corresponding parental SCs revealed interesting patterns in the gene expression heat map (Fig. 1A). First, the hiPSCs were clearly distinguishable from their respective parental SCs. This finding was verified by another clustering method with a distinct technique (see additional file 3: Clustering of the gene expression data with another method). Second, the gene expression profiles of the four hiPSC lines were linked to those of their parental SCs, while these profiles of the hiPSCs from different passages were clustered more closely with each other, rather than with those of the hiPSCs from the corresponding parental SCs (Fig. 1A). In support of these findings, a Pearson correlation analysis demonstrated that the gene expression profiles of the hiPSCs from different passages were more closely related to each other than to the hiPSCs from the same parental SCs (Fig. 1B and see also additional file 4: Correlation coefficient matrix for all cells). Furthermore, the above relationship between the hiPSCs and the parental SCs was verified by estimating the classification accuracy by leave-one-out cross-validation (LOOCV) on the nearest-neighbor classifier, based on Pearson's correlation distance (see additional file 5: Cross validation of cell classification).

### Gene expression signature for hiPSCs descended from different parent SCs

**Figure 2:**
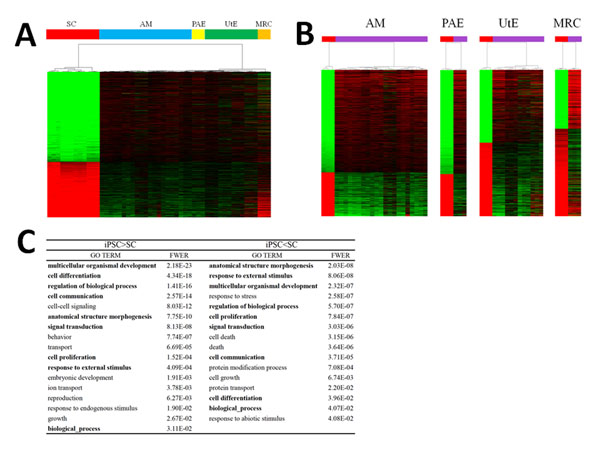
**Gene expression signature** (*A*) Heat map of 2,502 genes in the hiPSCs and parental cells. Cell types are indicated by colored bars. (*B*) Heat maps for the MRC, AM, UtE, and PAE hiPSCs and their corresponding parental cells. The hiPSCs and the parental SCs are discriminated by colored bars (red, SCs; purple, hiPSCs). (*C*) GO terms in the Biological Process, with the significance probabilities for the genes with higher and lower levels of expression. GO terms were summarized into 33 terms, as a macroscopic view, and the overlapped terms in the higher and lower expression classes are indicated by bold characters. See also additional file 6.

Analyses of the differences in gene expression between the four hiPSC lines and the parental SC lines revealed that 8,287 (out of 16,483) genes in the AM cells, 7,249 genes in the MRC cells, 7,465 genes in the PAE cells, and 6,314 genes in the UtE cells showed significant differences between the hiPSC lines and the corresponding parental lines, as determined using the Student’s *t*-test (for a false discovery rate [FDR] < 5% and requiring a ≥2.0-fold change in expression between the cells) (Fig. 2A). In total, 2,502 genes were categorized into a gene expression signature common to the above four gene sets with expression differences (Fig. 2B and see also additional files 6: Number matrix for common genes, and 7: List of 2,502 genes in the expression signature, together with the fold-changes in expression levels and FDR values).

In this expression signature, 62% of the genes (1,549 genes) were upregulated and 38% (953 genes) were downregulated in the hiPSCs, as compared to the parental SCs (Fig. 2B and see also additional file 7). From the 953 genes in the gene signature that were expressed at lower levels, gene ontology analyses revealed 60 terms with significant probability (family-wise error rate [FWER] <0.05), whereas the 1,549 genes that were expressed at higher levels were characterized by 89 terms (see additional file 8: List of enriched GO terms with significant probabilities (FWER < 0.05)). In total, 149 terms were found, and the GO analysis was determined to be inadequate for defining the biological functions of the expression signature in hiPSCs. The 149 terms were summarized into 33 terms as a macroscopic view; these terms shared 9 terms between the higher and lower expression levels (Fig. 2C).

### Network signature of hiPSCs by network screening

**Figure 3:**
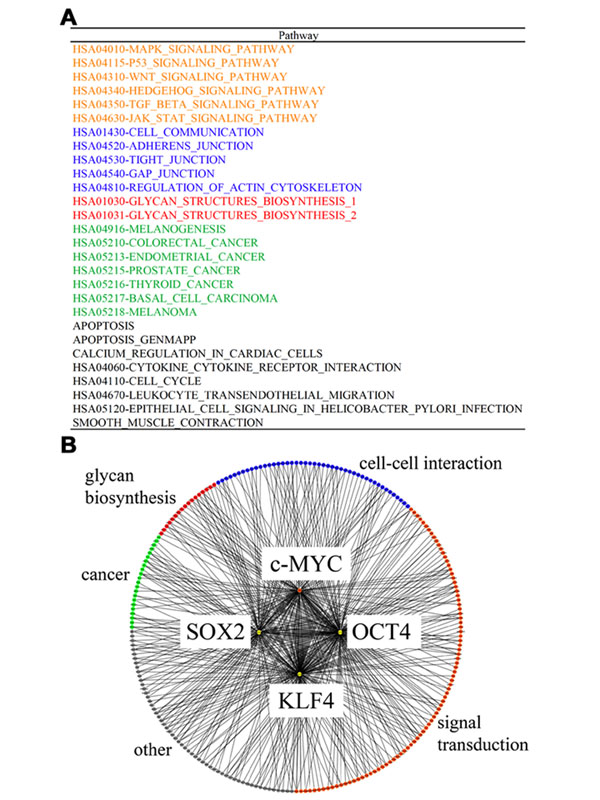
**Network signature** (*A*) List of network signatures. The pathways with significant probabilities are classified into the following categories: orange, pathways related to signal transduction; blue, pathways related to cell-cell interactions; red, pathways related to glycan biosynthesis; green, pathways related to cancer; and black, unclassified pathways. (*B*) Schematic presentation of networks. The four induced factors are described in the center, and the binding genes, which are colored according to the classification scheme described in (*A*), are connected by thin lines.

To elucidate the nature of the expression signature of the hiPSCs, we incorporated information on gene binding and function into a network analysis approach, named network screening [21] (see additional file 9: Schematic representation of the network screening used to obtain the network signature, and Methods). To prepare the network analysis, we identified 146 regulatory networks of 313 genes in the expression signature, which were classified with their functions using the gene sets defined previously [24] (see additional file 10: Reference networks and constituent genes, and Methods), among 519 genes that were identified as being bound by the four factors in ChIP-on-chip experiments [20]. We then analyzed the 146 reference networks, which were regarded as being directly induced by the four factors (OCT3/4, SOX2, KLF4, c-MYC), to define the network signature of the hiPSCs, according to the following two thresholds (Fig. 3): 1) the enrichment probability of the genes in the expression signature for each network; and 2) the consistency of the network structure in relation to the gene expression profile [21]. Thus, as the network signature, we defined 28 networks of 76 genes that fulfilled these conditions (Fig. 3A and see also additional file 11: Details of the network signature).

As expected, the network signature almost completely covered the pathways that were previously implicated in the reprogramming of hiPSC pluripotency (Figs. 3A and B ). For example, the relationship between reprogramming for pluripotency and signal transduction was emphasized for the TGF-β [25], Wnt [26], and MAPK pathways [27]. In addition, pathways related to cell-cell interactions were implicated. Although the molecular mechanisms underlying the cell-cell interactions in the inner cellular states are less understood, several studies have highlighted the importance of cellular communication through the extracellular matrix with respect to changes in the cellular states, such as those that occur during development and differentiation [28]. Furthermore, relationships to cancer-related pathways were identified, consistent with the fact that the four factors induce various cancer cells [29]; this finding may be useful in the prevention of cancer induction by hiPSCs. Although several pathways in the network signature remain to be characterized, it provides clues as to the molecular mechanisms underlying the reprogramming for hiPSC pluripotency and self-renewal, in contrast to the information obtained from a characterization based simply on GO terms.

### Networks with significant correlation between reprogramming and glycan biosynthesis

**Figure 4:**
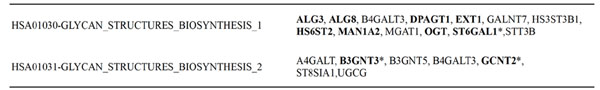
**Genes involved in two glycome biosynthesis pathways** The genes found in the expression signature are indicated by bold characters. Three genes related to glycan transfer are indicated by asterisks.

Interestingly, two regulatory networks related to the glycome, for linkage of the inner and outer cellular states, appeared in the network signature (Fig. 3A). In general, glycan biosynthesis is a multi-step process that requires a variety of enzymes, i.e., glycosyltransferases and enzymes involved in cytosolic sugar metabolism, and in many cases, glycan biosynthesis follows a glycan-specific, linear pathway. Most glycosyltransferases are regulated at the transcriptional level, thus warranting an assessment of the transcriptional profile of the glycan biosynthesis genes. In the two pathways, we found three genes (*ST6GAL1*, *B3GNT3*, and *GCNT2*) related to glycan transfer and two genes (*EXT1* and *HS6ST2*) related to heparan sulfate biosynthesis that were included in the expression signature (Fig. 4). These findings are consistent with recent studies that revealed the association between *N*-glycans and the maintenance of embryonic stem cell (ESC) pluripotency [5] and that between heparan sulfate and the reprogramming of ESCs [30]. Therefore, the genes identified in the above two pathways are candidates for the maintenance of the outer cellular state of iPSCs.

### Glycan signature unique to hiPSCs

**Figure 5:**
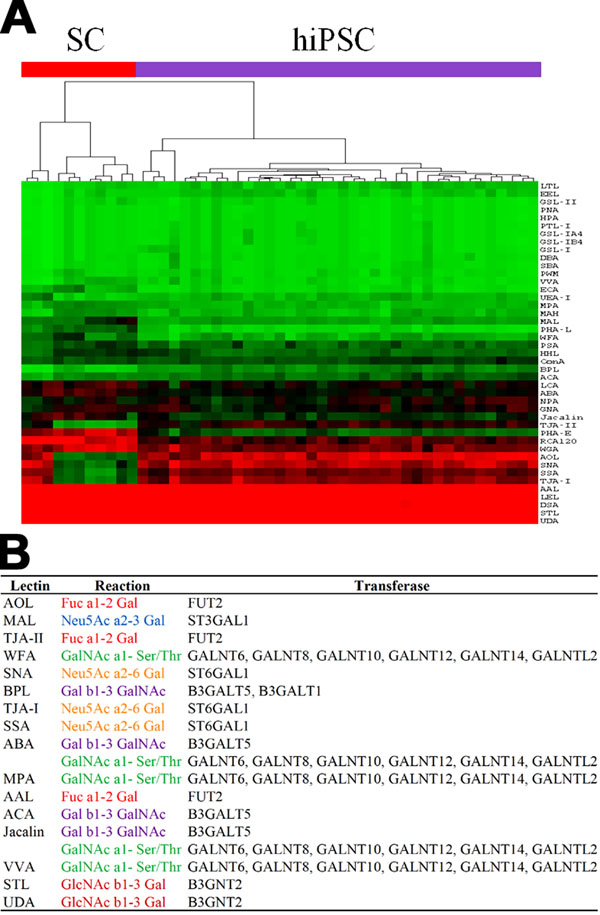
**Glycan signature** (*A*) Heat map and hierarchical clustering of lectins. The hiPSCs and parental SCs are depicted by colored bars (red, SCs; purple, hiPSCs). (*B*) Correspondence between lectin gene expression patterns and glycan signatures. The lectin-glycosyltransferase relationships are described, together with their reactions. The lectins were selected under the condition that the corresponding glycosyltransferases were found in the expression signature.

In addition to the expression and network signatures of the inner cell state, we examined the differences in the outer cellular states of the hiPSCs and the parental SCs using a lectin array, which detects glycan structures on cell surface proteins, based on glycan-lectin interactions [31]. In this analysis, the hiPSCs were clearly distinct from their parental SCs, and the dendrogram of the lectin microarray generated by unsupervised hierarchical clustering showed a clear separation between the hiPSCs and the parental SCs (Fig. 5A). Although the binding relationships between lectins and glycans and the relationships between the changes in glycan structures and the corresponding glycosyltransferases are redundant [32], we summarized the lectin-glycan-glycosyltransferase relationships using KEGG GLYCAN [33] and by manual curation of previous reports. We found strong correlations between the gene expression profiles of the glycosyltransferases and the corresponding lectin fluorescence intensities (see additional file 12: Lectin-glycan-glycosyltransferase relationships and correlations of lectin array intensities with glycosyltransferase expression patterns). This result indicates that the glycosyltransferases are coordinately expressed with the reprogramming, with the result that the hiPSCs bear glycan structures that are distinct from those of their parental SCs, reflecting the reprogramming of the inner cellular state.

Based on the Student’s *t*-test (FDR <0.05) analysis, 28 of the 43 lectins in the lectin microarray showed significant differences between the hiPSCs and the parental SCs (see also additional file 12). For the glycan signature, we assigned 16 lectins, which interacted with the 12 glycosyltransferases that were related to the six patterns of glycan reactions, based on the correspondence with the expression signature (Fig. 5B).

### Candidates of possible linkages between the inner and outer cellular states

Based on the correspondences between the expression and network signatures and between the expression and glycan signatures, we identified a total of 14 glycosyltransferases, since ST6GAL1 appeared in both sets of correspondences. These glycosyltransferases are potential candidates for the linkage between the inner and outer cellular states in hiPSCs. Interestingly, these glycosyltransferases may be related to the biosynthesis of a glycolipid that is characteristic of hiPSCs (see additional file 13: Knowledge-based relationships between glycosyltransferases and their biosynthetic pathways). Indeed, the allocation of the above glycosyltransferases to the pathways of “Glycan Biosynthesis and Metabolism” in KEGG GLYCAN (Table 1 and see also additional file 14: Locations of the glycosyltransferases detected in the present study in the pathways of “Glycan Biosynthesis and Metabolism”) revealed that the glycosyltransferases identified in the present study are important in the glycolipid biosynthetic pathway. We identified B3GALT5 in the biosynthetic pathway for the carbohydrate chains of the globo-series of glycosphingolipids bearing the well-known SSEA-3 and SSEA-4 epitopes for ESCs and iPSCs [34,35], and although FUT2 is not directly involved in the synthesis of these glycans, it was found in the neighboring pathway that leads to the type IV H antigen. Furthermore, B3GALT1 and GCNT2, in addition to B3GALT5 and FUT2, were found in the extensive biosynthetic pathway of the carbohydrate chains of the lacto- and neolacto-series glycosphingolipids that carry SSEA-1, which is intensively expressed in ESCs, but is absent in cells that have differentiated from ESCs [36]. In addition, the members of the GALNT family, responsible for the *O*-glycan biosynthetic pathway of sialyl-T antigen, which is the most abundant glycan in several carcinoma cell lines, and ST6GAL1 were only found in the *N*-glycan biosynthetic pathway, which is involved in the generation of cell-surface carbohydrate determinants and the differentiation antigens HB-6, CDw75, and CD76 [37]. These analyses identified the glycosyltransferases that are directly and indirectly related to known glycan epitopes, thereby indicating the key molecules and the marker epitopes involved in reprogramming.

**Table 1:** Relationships between glycosyltransferase expression, network, and glycan signature

Glycosyltransferase	Functions	Glycan structure
ST6GAL1	N-, O-Glycan and glycolipid biosynthesis	Siaa2,6Galb1,4GlcNAc-R
B3GNT3	O-Glycan biosynthesis	core1 extension
GCNT2	N-, O-Glycan and glycolipid biosynthesis	I antigen Siaa2,3Galb1,3GalNAca1-
ST3GAL1	O-Glycan biosynthesis	Ser/Thr
FUT2	N-, O-Glycan and glycolipid biosynthesis	H antigen
GALNT6	O-Glycan biosynthesis	GalNAca1-Ser/Thr
GALNT8	O-Glycan biosynthesis	GalNAca1-Ser/Thr
GALNT10	O-Glycan biosynthesis	GalNAca1-Ser/Thr
GALNT12	O-Glycan biosynthesis	GalNAca1-Ser/Thr
GALNT14	O-Glycan biosynthesis	GalNAca1-Ser/Thr
GALNTL2	Unknown	
B3GALT5	N-, O-Glycan and glycolipid biosynthesis	Galb1,3GlcNAc-R, SSEA-3
B3GALT1	N-, O-Glycan and glycolipid biosynthesis N- and O-Glycan, keratan sulfate	Galb1,3GlcNAc-R
B3GNT2	biosynthesis	polylactosamine

The fourteen glycosyltransferases with identified correspondences between expression, network, and glycan signatures were allocated to biosynthetic pathways, using the KEGG GLYCAN database with modifications. The names of the pathways are listed. See also additional file 12 for the detailed pathways of notable glycosyltransferases, according to the KEGG GLYCAN database.

### Further remarks on the present study

We analyzed more than 50 hiPSCs that were originally established from parental SCs, and the correspondence between each hiPSC and its parental SC was strictly controlled, which supports the present results based on a comparison with a clear genetic relationship. To further clarify the molecular mechanisms of the pluripotency, embryonic stem cells (ESCs) should be analyzed, following the context of the present study. Indeed, the pluripotency of hiPSCs has been extensively evaluated with reference to that of human ESCs, by various comparisons [38-43]. At present, we have prepared more than 100 hiPSCs with higher passages, and their comparisons with ESCs will be reported in the near future.

As for the experimental measurements, two types of data, gene expression and glycan structure, were analyzed by using microarrays and lectin arrays in the present study. To comprehensively understand the features of hiPSCs, more experimental data should be utilized, such as DNA-methylation and mi-RNA data. In particular, the recent availability of the next-gen sequencer will produce RNA-seq and ChIP-seq data with more accurate measurements of gene expression and concrete information about the regulated genes. In addition, vast amounts of protein interaction data are accumulating. A comprehensive analysis integrating the various data from more hiPSCs will be reported in the near future.

## Conclusions

The present study is the first to reveal the relationships between gene expression patterns and cell surface changes in hiPSCs, and it reinforces the importance of the cell surface to identify established iPSCs from SCs. In addition, given the variability of iPSCs, which is related to the characteristics of the parental SCs, a glycosyltransferase expression assay should be established that allows more precise definition of hiPSCs and facilitates their standardization, which are important steps towards eventual therapeutic applications of hiPSCs.

## Methods

### Cell experiments

Somatic cell pellets were harvested by scraping. The hiPSCs were incubated at 37°C, in a solution containing 1 mg/ml collagenase IV (Invitrogen, Carlsbad, CA), 1 mM CaCl_2_, 20% KNOCKOUT^TM^ Serum Replacement (KSR), and 10% ACCUMAX (Innovative Cell Technologies, Inc., San Diego, CA). When the edges of the colonies started to dissociate from the bottom of the dish, the collagenase solution was removed and the cells were washed with medium. Colonies were then picked up and collected.

MRC-5 and amniotic mesodermal (AM) cells were maintained in POWEREDBY 10 medium (MED Shiratori Co., Ltd., Tokyo, Japan). The human placental artery endothelial (PAE) cells were harvested from human placenta. To isolate the arterial endothelium, we used the explant culture method, in which the cells were outgrown from pieces of the placenta’s arterial vessels. Briefly, arterial vessels were separated from arteries in the chorionic plate, and chopped into approximately 5-mm^3^ pieces. The pieces were washed in endothelial basal medium-2 (EBM-2; Cambrex, Walkersville, MD) and cultured in EGM-2MV medium (Cambrex), which consisted of EBM-2, 5% fetal bovine serum (FBS), and the supplemental growth factors VEGF, bFGF, EGF, and IGF. The arterial vessels attached to the substrata of the culture dishes (BD Falcon; Becton Dickinson, San Jose, CA). Cells migrated out from the surface of the tissues after about 20 days of incubation at 37°C in 5% CO_2_. The cells were harvested in PBS containing 0.1% trypsin and 0.25 mM EDTA, and were re-seeded at a density of 3 × 10^5^ cells in a 10-cm dish. Confluent monolayers of cells were subcultured. The culture medium was replaced every 3-4 days. Human uterine endometrium (UtE) was harvested from a patient with endometriosis. The endometrium was sterilized in PBS and cut into small pieces with dissection scissors. These pieces were placed in Dulbecco’s Modified Eagle’s Medium (Sigma Chemical Co. St. Louis, MO), supplemented with 10% FBS and an antibiotic-antimycotic (100×) solution (Invitrogen), and incubated for 10-14 days at 37°C in a humidified 5% CO_2_ atmosphere. Subconfluent adherent cells were harvested in PBS containing 0.06% trypsin and 0.005% EDTA, and were subcultured. The culture medium was replaced every 4 days. This study was approved by the Ethical Committee of the National Institute for Child Health and Development. The purpose of this study was explained thoroughly to the patients, who gave their written informed consent.

hiPSCs were cultivated on irradiated MEFs in iPSellon medium (Cardio, Osaka, Japan), supplemented with 10 ng/ml recombinant human bFGF (Wako Pure Chemicals, Osaka, Japan). hiPSCs were established from MRC-5 and AM cells, as previously described [21,22]. In addition, hiPSCs were established from PAE and UtE cells in the present study. Briefly, 1 × 10^5^ cells were infected overnight with pooled viral supernatants, obtained by the transfection of HEK293FT cells (TransIT-293 reagent; Mirus, Madison, WI) with the retroviral vector pMXs, which encodes the cDNAs for OCT3/4, SOX2, KLF4, and c-MYC, together with the packaging plasmids pCLGagPol and pHCMV-VEV-G (a gift from T. Kiyono, National Cancer Center Research Institute, Tokyo, Japan). Four days after infection, the cells were split, plated on irradiated MEFs in 100-mm dishes, and maintained in iPSellon medium until colonies formed.

The immunocytochemical analysis was performed as described previously [22,23]. Human cells were fixed with 4% paraformaldehyde in PBS for 10 min at 4 °C. After washing with 0.1% Triton X-100 in PBS (PBST), the cells were prehybridized in blocking buffer for 1–12 h at 4 °C, and then incubated for 6–12 h at 4°C with the following primary antibodies: anti-SSEA4 (1 : 300 dilution; Chemicon, Temecula, CA), anti-TRA-1–60 (1 : 300; Chemicon), anti-Oct4 (1 : 50; Santa Cruz Biotechnology, Santa Cruz, CA), anti-Nanog (1 : 300; ReproCELL, Tokyo, Japan), and anti-Sox2 (1 : 300; Chemicon). The cells were then incubated with anti-rabbit IgG, anti-mouse IgG or anti-mouse IgM conjugated with Alexa Fluor 488 or Alexa Fluor 546 (1: 500; Molecular Probes, Eugene, OR) in blocking buffer for 1 h at room temperature. The cells were counterstained with DAPI, and then mounted using a SlowFade light antifade kit (Molecular Probes).

Teratoma formation was performed as described previously [22,23]. The 1:1 mixtures of the AM-hiPSC suspension and Basement Membrane Matrix (BD Biosciences, San Jose, CA) were implanted subcutaneously, at 1.0 × 10^7^ cells / site, into immunodeficient, non-obese diabetic (NOD)/severe combined immunodeficiency (SCID) mice (CREA, Tokyo, Japan). Teratomas were surgically dissected out 6–10 weeks after implantation, and were fixed with 4% paraformaldehyde in PBS and embedded in paraffin. Sections of 10-μm thickness were stained with hematoxylin-eosin.

### Gene expression analysis

Total RNA samples were extracted using ISOGEN (NipponGene). The global gene expression patterns and changes in mRNA levels were monitored using Agilent Whole Human Genome Microarray chips (G4112F) with one-color (Cyanine 3) dye. This microarray chip covers 41,000 well-characterized human genes and transcripts. The raw microarray data were submitted to the GEO (Gene Expression Omnibus) microarray data archive (http://www.ncbi.nlm.nih.gov/geo/) at the NCBI (accession number: GSE 20750). After background correction using a Normal plus Exponential convolution model, which adjusts the foreground to the background, we used an offset to dampen the variation of the log-ratios for intensities close to zero.

Among the 41,000 probes, 16,483 representative probes corresponding to MAQC unique genes were used for the following analyses [44]. Global array clustering was performed by the complete linkage method with Euclidean distance, and was visualized using the Java TreeView 1.1.0 software; the gene expression values are displayed as normalized log ratios. Cell line similarities were measured using Pearson correlation coefficients. To further validate whether the global gene expression is different in each origin cell, we evaluated the classification accuracy by leave-one-out cross-validation (LOOCV) on the nearest-neighbor classifier, based on Pearson's correlation distance. To obtain the expression signatures, we performed a differential analysis for each origin cell: differences between the two arbitrary datasets were evaluated by the Student’s t-test for the expression of each gene. Thereafter, the false discovery rate (FDR) was estimated using the Benjamini–Hochberg procedure. Differentially expressed genes were selected if they satisfied both FDR <0.05 and a 2.0-fold change in the average values for the cell lines being compared. The gene ontology analysis was performed using the GO Term Finder Perl script [45] (http://go.princeton.edu/cgi-bin/GOTermFinder), with EBI human GO annotations and generic GO slim annotations (http://www.geneontology.org/).

### Network screening

Network screening was performed as described previously [21]. This analysis is based on the procedure for estimating the consistency of a network structure (directed acyclic graph) with the measured data for the constituent variables in the graph. The joint density function for a given network (reference network) was recursively factorized into conditional density functions, according to the parent-child relationship in the graph. The conditional functions were quantified into log-likelihoods, using linear regression for the measured data, with the assumption that the data followed a normal distribution. The probability of the log-likelihood for the network structure (graph consistency probability; GCP) was then estimated from the distribution of log-likelihoods for 2,000 networks, generated under the condition that the networks shared the same numbers of nodes and edges as those of the given network. The significance probability of the given network was set at 0.05 in this analysis.

In the present study, the GCP was estimated for the ensemble of reference networks, to extract the candidate activated networks in the hiPSCs, in a process termed ‘network screening’. The reference networks were constructed using the ChIP-on-Chip data and the classification scheme for gene function. The genes bound by four factors were cited from a previous report [20], and were divided into sub-networks according to the functional gene sets previously defined in the Molecular Signatures Database (MSigDB) [24]. The sub-networks that included at least one gene of the expression signature were then selected. The set of selected sub-networks was used as the reference network for network screening.

### Glycan analysis

We analyzed cell surface glycans with a lectin microarray [31]. The 43 lectins were dissolved at a concentration of 0.5 mg/ml in spotting solution (Matsunami Glass, Osaka, Japan), and were spotted onto epoxysilane-coated glass slides (Nexterion Slide E Epoxysilane-coated Substrate 25 × 75.6 × 1 mm; Schott, Mainz, Germany) attached to a silicone rubber sheet, using a non-contact microarray printing robot (MicroSys 4000; Genomic Solutions, Ann Arbor, MI). The lectins were spotted in triplicate, with a spot diameter of 500 μm. The glass slides were incubated at 25°C for 3 h, to allow lectin immobilization. The lectin-immobilized glass slides were then washed with probing buffer (25 mM Tris-HCl [pH 7.5], 140 mM NaCl, 2.7 mM KCl, 1 mM CaCl2, 1 mM MnCl2, 1% [v/v] Triton X-100), and incubated with the blocking reagent N102 (NOF, Tokyo, Japan) at 20°C for 1 h. Finally, the lectin-immobilized glass slides were flooded with TBS containing 0.1% NaN_3_ and stored at 4°C. The cell membrane faction was prepared using the CelLytic MEM Protein Extraction Kit (Sigma-Aldrich, Tokyo, Japan), and the protein concentration was determined using the MicroBCA Protein Assay Reagent kit (Thermo Fisher Scientific, Waltham, MA). After dilution in PBST (10 mM PBS [pH 7.4], 140 mM NaCl, 2.7 mM KCl, 1% Triton X-100), the cell membrane fraction was labeled with Cy3 NHS ester (GE Healthcare Ltd., Buckinghamshire, England). After dilution in probing buffer to the desired concentration, the Cy3-labeled cell membrane fraction was applied to the lectin microarray and incubated at 20°C overnight. After washing with the probing buffer, fluorescence images were acquired using an evanescent-field activated fluorescence scanner (SC-Profiler; GP BioScience, Kanagawa, Japan). The fluorescence signal of each spot was quantified using the Array Pro Analyzer ver. 4.5 software (Media Cybernetics, Bethesda, MD), and the background value was subtracted. The values shown for the lectin signals represent the average of triplicate spots.

## Competing interests

The authors declare that they have no competing interests.

## Authors' contributions

S.S. (computational analysis and manuscript preparation), Y.O. (cell experiments and DNA microarray), Y.I. (DNA microarray and manuscript preparation), H.T. (lectin microarray and manuscript preparation), M.T. (cell experiments and manuscript preparation), H.A. (cell experiments), K.N. (cell experiments), E.C. (cell experiments), Y.F. (cell experiments), Y.M. (vector construction), H.O. (vector construction), N.K. (vector construction), Y.S. (lectin microarray), A.U. (supervision of cell experiments), J.H. (lectin microarray), K.H. (computational analysis and manuscript preparation), and M.A. (project leader and coordination).

## Additional files

There are 14 additional files in the present analysis. For convenience, we provide an overview of the additional files. Additional files 1, 2, 3, 4, 5 are related to the cell classification in Figure 1: the details of the cell lines and their experimental establishment are described in files 1 and 2, and the details of the analyses of the expression data are described in files 3-5. Additional figures 6-8 are related to the gene expression signature in Figure 2: the details of the analyzed data are described in files 6 and 7, and the results obtained by a standard analysis are described in file 8. Additional files 9, 10, 11 are related to the network signature in Figure 3: the methodological aspects of the network screening are described in files 9 and 10, and in file 11, the detailed results are presented. Additional files 12, 13, 14 are related to the glycan signature in Figure 5: all of the information for interpreting the analyzed results is presented in the three files.

## Supplementary Material

Additional file 1 **Cell lines and numbers of passages analyzed in the present study.** The following abbreviations are used for the human somatic cell (SC) and induced pluripotent stem cell (hiPSC) sources: AM, amniotic membrane; PAE, placental artery endothelial; UtE, uterine endometrium; and MRC, MRC-5 cell line. The AM and MRC cell lines were named previously [22,23]. The number of passages for each cell line is indicated by the letter ‘p’ followed by an Arabic number. Click here for file

Additional file 2 **Generation of iPSCs from human PAE cells**. (*A*) PAE cells from the arterial endothelium of a human placenta (a), and generation of hiPSCs through epigenetic reprogramming by retrovirus infection-mediated expression of OCT4, SOX2, KLF4, and c-MYC (b). (*B*) Expression patterns of the pluripotent cell markers, TRA-1-60, SSEA-4, NANOG, OCT3/4, and SOX2. The cell nuclei were stained with DAPI. (*C*) Hematoxylin-eosin staining of sections of teratomas generated by PAE-hiPSC implantation. The histological examination revealed that the tumors contain neural tissues (a: ectoderm), cartilage (b: mesoderm), and a gut-like epithelial tissue (c: endoderm). Click here for file

Additional file 3 **Clustering for all cells by another method**. Another clustering was performed by the WARD method, instead of the complete linkage method of Figure 1, with Euclidean distance, and was visualized using the Java TreeView 1.1.0 software. The gene expression values are displayed as normalized log ratios. The abbreviations used are the same as those listed in Figure 1 and additional file 1. Click here for file

Additional file 4 **Correlation coefficient matrix for all cells**. Pearson’s correlation coefficients between 51 cells for the expression profiles of all genes were calculated. The abbreviations used are the same as those listed in Figure 1 and additional file 1. Click here for file

Additional file 5 **Cross-validation of cell classification**. The classification accuracy was evaluated by leave-one-out cross-validation (LOOCV) on the nearest-neighbor classifier, based on the Pearson's correlation distance. Click here for file

Additional file 6 **Number matrix for common genes**. The numbers of genes that were different between the iPSCs and SCs are listed on the diagonal of the matrix, and those that were shared between the four gene sets that showed expression differences between the iPSCs are listed above the diagonal. The abbreviations used are the same as those listed in Figure 1. Click here for file

Additional file 7 **List of 2,502 genes in the expression signature, together with the fold-changes in expression levels and FDR values**. The fold-change values are listed for the minimum values among the four sets of comparisons between iPSCs and SCs (+, iPSCs>SCs; -, iPSCs<SCs), and the FDR values shown are the maximum values among these sets. Click here for file

Additional file 8 List of enriched GO terms with significant probabilities (FWER < 0.05). Click here for file

Additional file 9 **Schematic representation of the procedure used to obtain the network signature**. The procedure for obtaining the network signature from the expression signature is shown schematically. The detailed procedure is as follows: 1) We first prepare the information for the gene sets to which the transcriptional factors bind, as deduced from the ChIP-on-chip experiments [20]; 2) Next, we prepare the information for the gene sets that were classified using knowledge of biological functions [24]; 3) The large gene sets in step 1 are divided into smaller subsets, according to the classification scheme of the gene sets in step 2; 4) If at least one gene in the expression signature is included in each gene subset in step 3, then the subset is regarded as a reference network; 5) In each reference network, the enrichment probability of the genes in the expression signature is tested with a significance probability of 0.05. Thus, we narrow down the network signature from the reference networks, in terms of gene numbers; 6) The significant reference networks identified in step 5 are further tested by calculating the graph consistency probability, which assesses the consistency between the network structure and the expression data for the constituent genes [24]. In this step, we further refine the network signature, in terms of both the network structure and the extent of gene expression; 7) Finally, we define the network signature, using the reference networks that passed the tests in steps 5 and 6. Click here for file

Additional file 10 Reference networks and constituent genes. Click here for file

Additional file 11 **Details of the network signature**. The characters in the above list are colored, according to the classification of biological function shown in Figure 2A. Click here for file

Additional file 12 **Lectin-glycan-glycosyltransferase relationships and correlations of lectin array intensities with glycosyltransferase expression patterns**. Lectins with FDR<0.05 are colored red. The glycosyltransferases in the expression signature are indicated by a circle in the column "Expression signature" in "Gene expression". The Pearson's correlation coefficients between the lectin signal intensities and the expression profiles of the corresponding glycosyltransferases are listed, together with the significance probabilities. The original lectin array data can be obtained by request to HT or JH. Click here for file

Additional file 13 Knowledge-based relationships between glycosyltransferases and their biosynthetic pathways. Click here for file

Additional file 14 **Locations of the glycosyltransferases detected in the present study in the pathways of “Glycan Biosynthesis and Metabolism”**. The glycosyltransferases listed in Table 1 were allocated to the pathways in “1.7 Glycan Biosynthesis and Metabolism” of the KEGG GLYCAN program (http://www.genome.jp/kegg/pathway.html#glycan). The glycosyltransferases and epitopes related to differentiation are indicated by red-colored boxes and red lines, respectively, in each pathway (see the text for details). Click here for file
