# Supplementary material for: Possible linkages between the inner and outer cellular states of human induced pluripotent stem cells
Source: BMC Syst Biol. 2011 Jun 20;5(Suppl 1):S17. doi: 10.1186/1752-0509-5-S1-S17 (PMC3121117; doi:10.1186/1752-0509-5-S1-S17)
Supplement: Additional file 1 — Cell lines and numbers of passages analyzed in the present study. The following abbreviations are used for the human somatic cell (SC) and induced pluripotent stem cell (hiPSC) sources: AM, amniotic membrane; PAE, placental artery endothelial; UtE, uterine endometrium; and MRC, MRC-5 cell line. The AM and MRC cell lines were named previously [22,23]. The number of passages for each cell line is indicated by the letter ‘p’ followed by an Arabic number. [file 1752-0509-5-S1-S17-S1.doc]

**Additional file 1: Cell lines and numbers of passages analyzed in the present study.**

The following abbreviations are used for the human somatic cell (SC) and induced pluripotent stem cell (hiPSC) sources: AM, amniotic membrane; PAE, placental artery endotherial; UtE, uterine endometrium; and MRC, MRC-5 cell line. The AM and MRC cell lines are named according to the previous reports [22, 23]. The numbers of passages of each cell line are indicated by the letter ‘p’ followed by an Arabic numbers.
