# Supplementary material for: Possible linkages between the inner and outer cellular states of human induced pluripotent stem cells
Source: BMC Syst Biol. 2011 Jun 20;5(Suppl 1):S17. doi: 10.1186/1752-0509-5-S1-S17 (PMC3121117; doi:10.1186/1752-0509-5-S1-S17)
Supplement: Additional file 2 — Generation of iPSCs from human PAE cells. (A) PAE cells from the arterial endothelium of a human placenta (a), and generation of hiPSCs through epigenetic reprogramming by retrovirus infection-mediated expression of OCT4, SOX2, KLF4, and c-MYC (b). (B) Expression patterns of the pluripotent cell markers, TRA-1-60, SSEA-4, NANOG, OCT3/4, and SOX2. The cell nuclei were stained with DAPI. (C) Hematoxylin-eosin staining of sections of teratomas generated by PAE-hiPSC implantation. The histological examination revealed that the tumors contain neural tissues (a: ectoderm), cartilage (b: mesoderm), and a gut-like epithelial tissue (c: endoderm). [file 1752-0509-5-S1-S17-S2.doc]

**
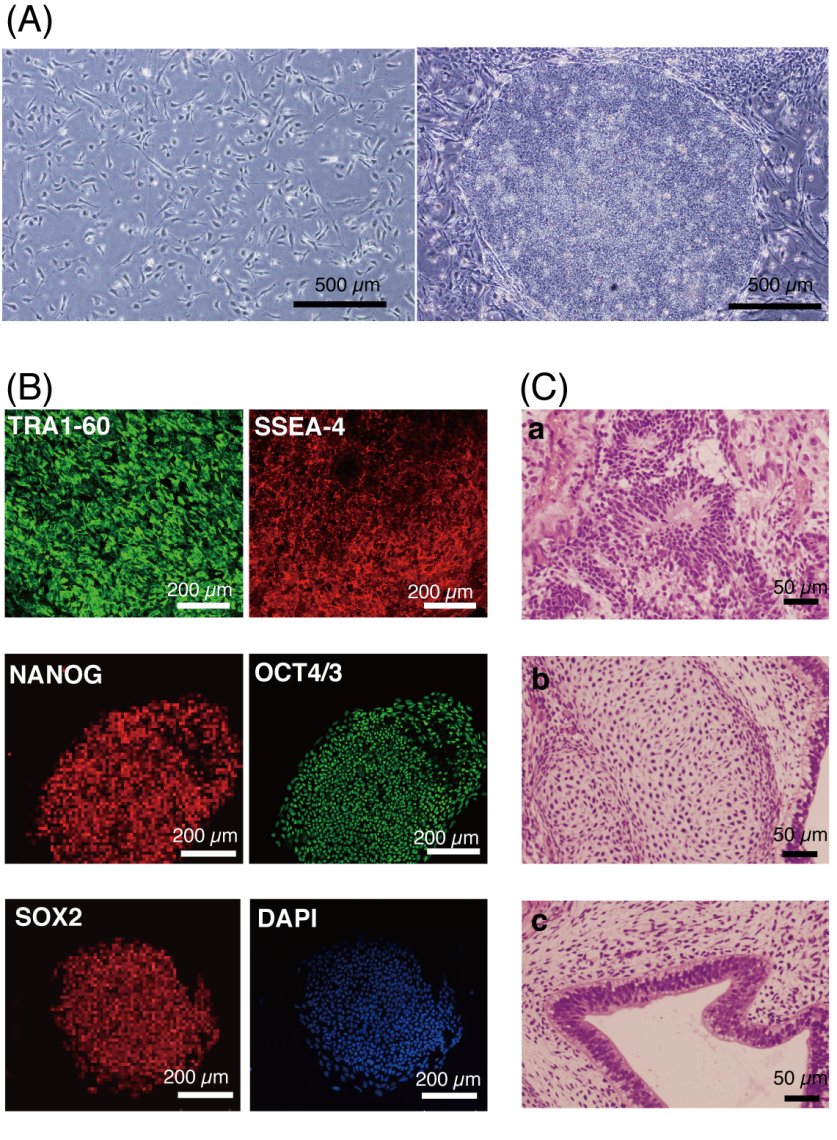
**

**Additional file 2: Generation of iPSCs from human PAE cells.**

(*A*) PAE cells from the arterial endothelium of a human placenta (a), and generation of hiPSCs through epigenetic reprogramming by retrovirus infection-mediated expression of OCT4, SOX2, KLF4, and c-MYC (b). (*B*) Expression patterns of the pluripotent cell markers, TRA-1-60, SSEA-4, NANOG, OCT3/4, and SOX2. The cell nuclei are stained with DAPI. (*C*) Hematoxylin-eosin staining of sections of teratomas generated by PAE-hiPSC implantation. Histological examination reveals that the tumors contain neural tissues (a: ectoderm), cartilage (b: mesoderm), and a gut-like epithelial tissue (c: endoderm).
