# Supplementary figures and images for: Possible linkages between the inner and outer cellular states of human induced pluripotent stem cells
Source: BMC Syst Biol. 2011 Jun 20;5(Suppl 1):S17. doi: 10.1186/1752-0509-5-S1-S17 (PMC3121117; doi:10.1186/1752-0509-5-S1-S17)

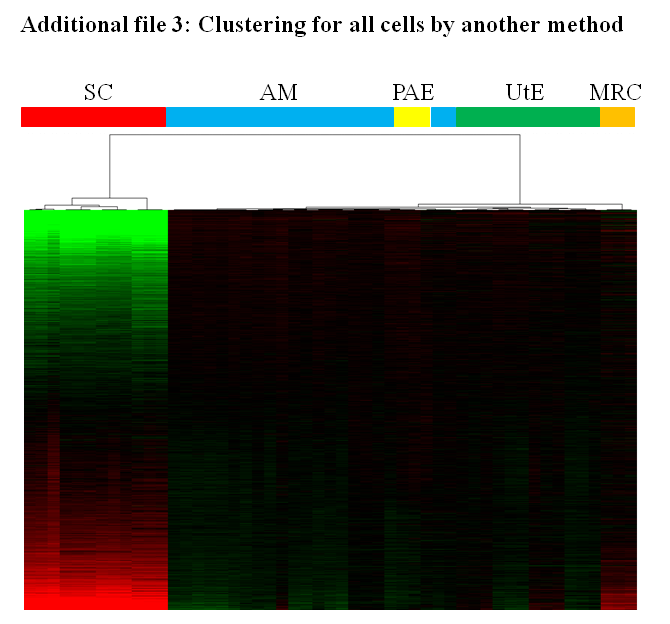

Supplement: Additional file 3 — Clustering for all cells by another method. Another clustering was performed by the WARD method, instead of the complete linkage method of Figure 1, with Euclidean distance, and was visualized using the Java TreeView 1.1.0 software. The gene expression values are displayed as normalized log ratios. The abbreviations used are the same as those listed in Figure 1 and additional file 1. [file 1752-0509-5-S1-S17-S3.tif]
