# Supplementary material for: Possible linkages between the inner and outer cellular states of human induced pluripotent stem cells
Source: BMC Syst Biol. 2011 Jun 20;5(Suppl 1):S17. doi: 10.1186/1752-0509-5-S1-S17 (PMC3121117; doi:10.1186/1752-0509-5-S1-S17)
Supplement: Additional file 4 — Correlation coefficient matrix for all cells. Pearson’s correlation coefficients between 51 cells for the expression profiles of all genes were calculated. The abbreviations used are the same as those listed in Figure 1 and additional file 1. [file 1752-0509-5-S1-S17-S4.doc]

**
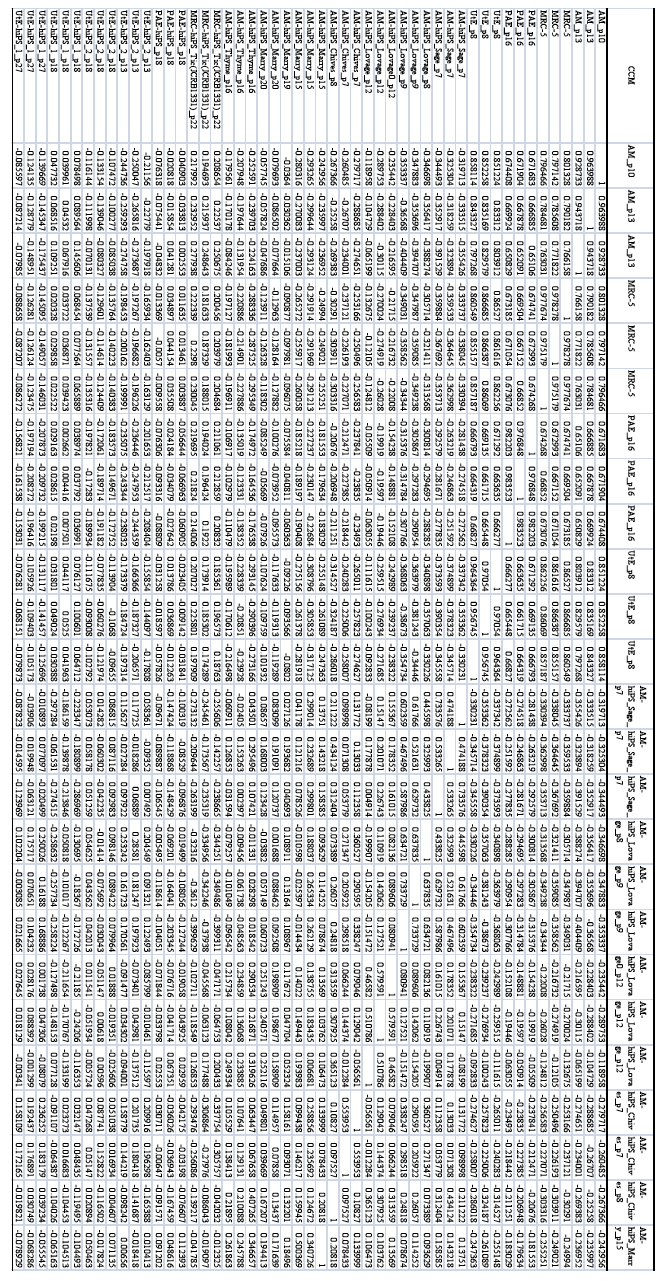
 (to be continued)**


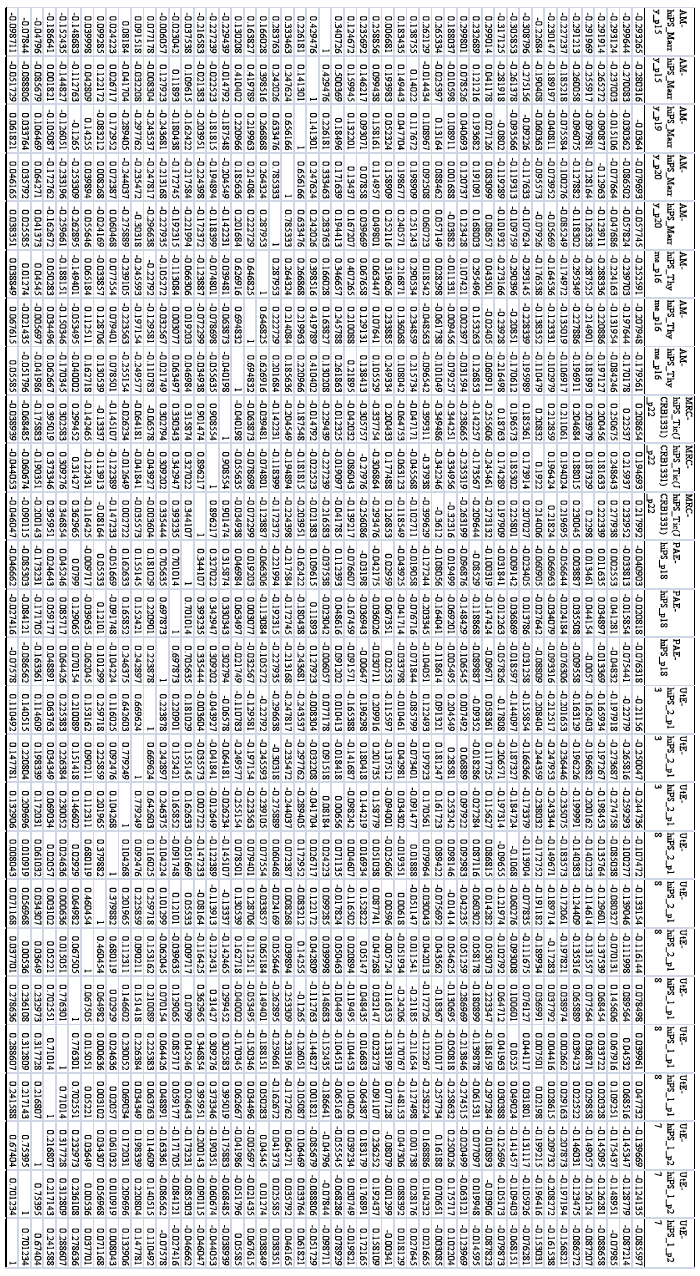


**Additional file 4: Correlation coefficient matrix for all cells.**

Pearson’s correlation coefficients between 51 cells for the expression profiles of all genes were calculated. The abbreviations used are the same as those listed in Figure 1 and Additional file 1.
