# Supplementary material for: Possible linkages between the inner and outer cellular states of human induced pluripotent stem cells
Source: BMC Syst Biol. 2011 Jun 20;5(Suppl 1):S17. doi: 10.1186/1752-0509-5-S1-S17 (PMC3121117; doi:10.1186/1752-0509-5-S1-S17)
Supplement: Additional file 5 — Cross-validation of cell classification. The classification accuracy was evaluated by leave-one-out cross-validation (LOOCV) on the nearest-neighbor classifier, based on the Pearson's correlation distance. [file 1752-0509-5-S1-S17-S5.doc]

**Additional file 5 – Cross-validation of cell classification**

The query sample is denoted in the first column, and the sample with the highest correlation coefficient value is denoted in the second column, with its value in the third column.
