# Supplementary material for: Possible linkages between the inner and outer cellular states of human induced pluripotent stem cells
Source: BMC Syst Biol. 2011 Jun 20;5(Suppl 1):S17. doi: 10.1186/1752-0509-5-S1-S17 (PMC3121117; doi:10.1186/1752-0509-5-S1-S17)
Supplement: Additional file 6 — Number matrix for common genes. The numbers of genes that were different between the iPSCs and SCs are listed on the diagonal of the matrix, and those that were shared between the four gene sets that showed expression differences between the iPSCs are listed above the diagonal. The abbreviations used are the same as those listed in Figure 1. [file 1752-0509-5-S1-S17-S6.doc]

**Additional file 6: Number matrix for common genes**

The numbers of genes that were different between the iPSCs and SCs are listed on the diagonal of the matrix, and those that were shared between the four gene sets that showed expression differences between the iPSCs are listed above the diagonal. The abbreviations used are the same as those listed in Figure 1.
