# Supplementary material for: Possible linkages between the inner and outer cellular states of human induced pluripotent stem cells
Source: BMC Syst Biol. 2011 Jun 20;5(Suppl 1):S17. doi: 10.1186/1752-0509-5-S1-S17 (PMC3121117; doi:10.1186/1752-0509-5-S1-S17)
Supplement: Additional file 9 — Schematic representation of the procedure used to obtain the network signature. The procedure for obtaining the network signature from the expression signature is shown schematically. The detailed procedure is as follows: 1) We first prepare the information for the gene sets to which the transcriptional factors bind, as deduced from the ChIP-on-chip experiments [20]; 2) Next, we prepare the information for the gene sets that were classified using knowledge of biological functions [24]; 3) The large gene sets in step 1 are divided into smaller subsets, according to the classification scheme of the gene sets in step 2; 4) If at least one gene in the expression signature is included in each gene subset in step 3, then the subset is regarded as a reference network; 5) In each reference network, the enrichment probability of the genes in the expression signature is tested with a significance probability of 0.05. Thus, we narrow down the network signature from the reference networks, in terms of gene numbers; 6) The significant reference networks identified in step 5 are further tested by calculating the graph consistency probability, which assesses the consistency between the network structure and the expression data for the constituent genes [24]. In this step, we further refine the network signature, in terms of both the network structure and the extent of gene expression; 7) Finally, we define the network signature, using the reference networks that passed the tests in steps 5 and 6. [file 1752-0509-5-S1-S17-S9.doc]

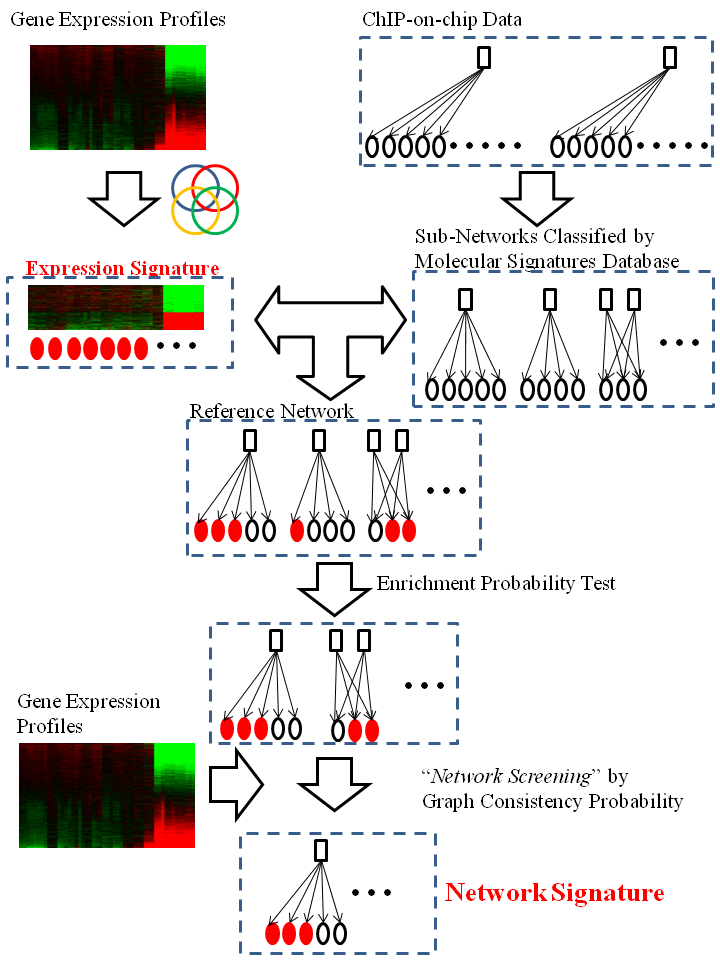


**Additional file 9: Schematic representation of network screening used to obtain the network signature.**

The network screening for obtaining the network signature from the expression signature is shown schematically. The detailed procedure is as follows:

1) We first prepare the information on the gene sets to which the transcriptional factors bind, as deduced from the ChIP-on-chip experiments [20];

2) Next, we prepare the information on the gene sets that were classified using knowledge of biological functions [24];

3) The large gene sets in step 1 are divided into smaller subsets, according to the classification scheme of the gene sets in step 2;

4) If at least one gene in the expression signature is included in each gene subset in step 3, then the subset is regarded as a reference network;

5) In each reference network, the enrichment probability of the genes in the expression signature is tested with a significance probability of 0.05. Thus, we narrow down the network signature from the reference networks, in terms of gene numbers;

6) The significant reference networks identified in step 5 are further tested by calculating the graph consistency probability, which assesses the consistency between the network structure and the expression data for the constituent genes [21]. In this step, we further refine the network signature, in terms of both the network structure and the extent of gene expression;

7) Finally, we define the network signature using the reference networks that passed the tests in steps 5 and 6.
