# Supplementary material for: Possible linkages between the inner and outer cellular states of human induced pluripotent stem cells
Source: BMC Syst Biol. 2011 Jun 20;5(Suppl 1):S17. doi: 10.1186/1752-0509-5-S1-S17 (PMC3121117; doi:10.1186/1752-0509-5-S1-S17)
Supplement: Additional file 13 — Knowledge-based relationships between glycosyltransferases and their biosynthetic pathways. [file 1752-0509-5-S1-S17-S13.doc]

**Additional file 13: Knowledge-based relationships between glycosyltransferases and their biosynthetic pathways**
