# Supplementary material for: Possible linkages between the inner and outer cellular states of human induced pluripotent stem cells
Source: BMC Syst Biol. 2011 Jun 20;5(Suppl 1):S17. doi: 10.1186/1752-0509-5-S1-S17 (PMC3121117; doi:10.1186/1752-0509-5-S1-S17)
Supplement: Additional file 14 — Locations of the glycosyltransferases detected in the present study in the pathways of “Glycan Biosynthesis and Metabolism”. The glycosyltransferases listed in Table 1 were allocated to the pathways in “1.7 Glycan Biosynthesis and Metabolism” of the KEGG GLYCAN program (http://www.genome.jp/kegg/pathway.html#glycan). The glycosyltransferases and epitopes related to differentiation are indicated by red-colored boxes and red lines, respectively, in each pathway (see the text for details). [file 1752-0509-5-S1-S17-S14.doc]

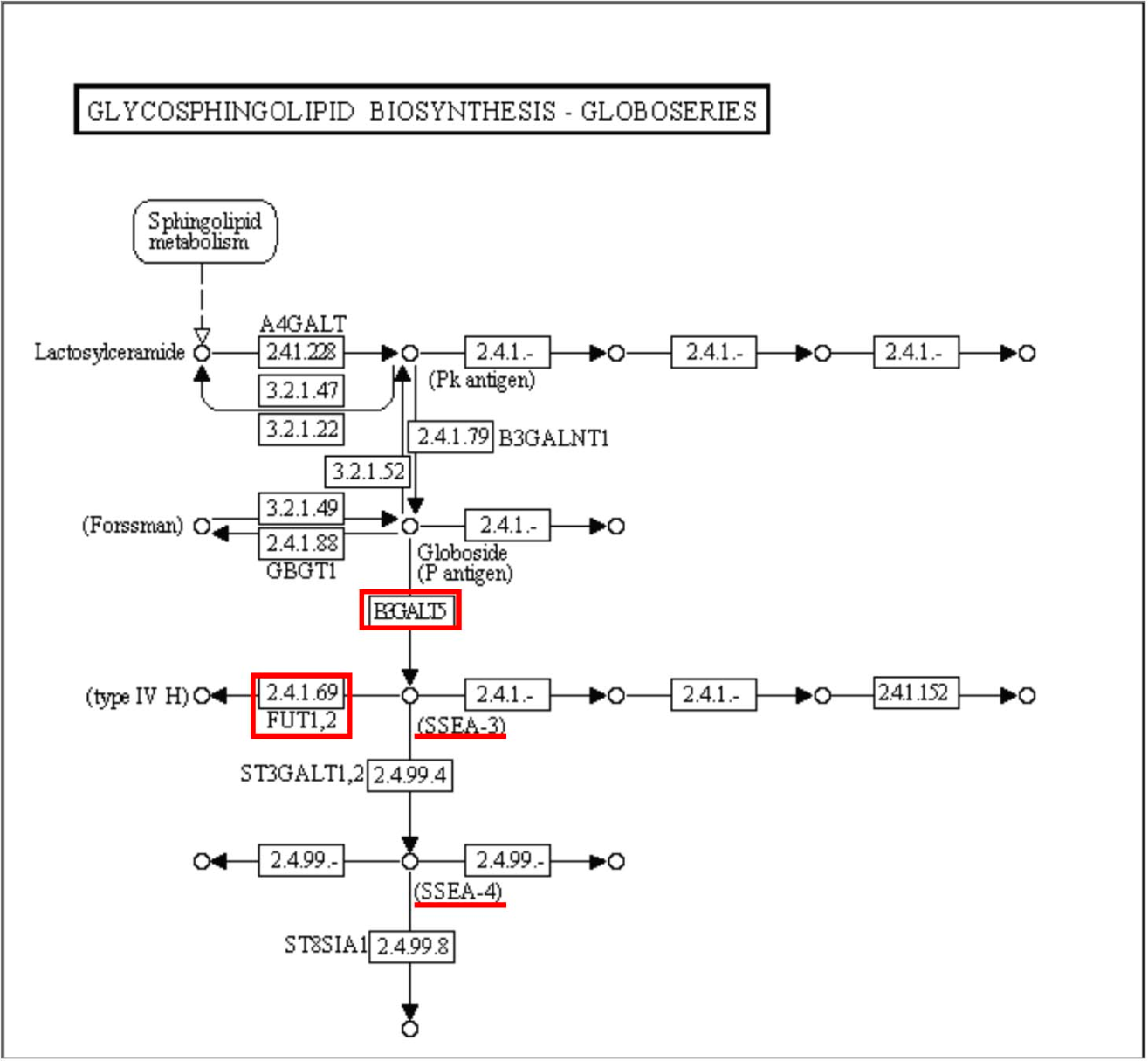


**
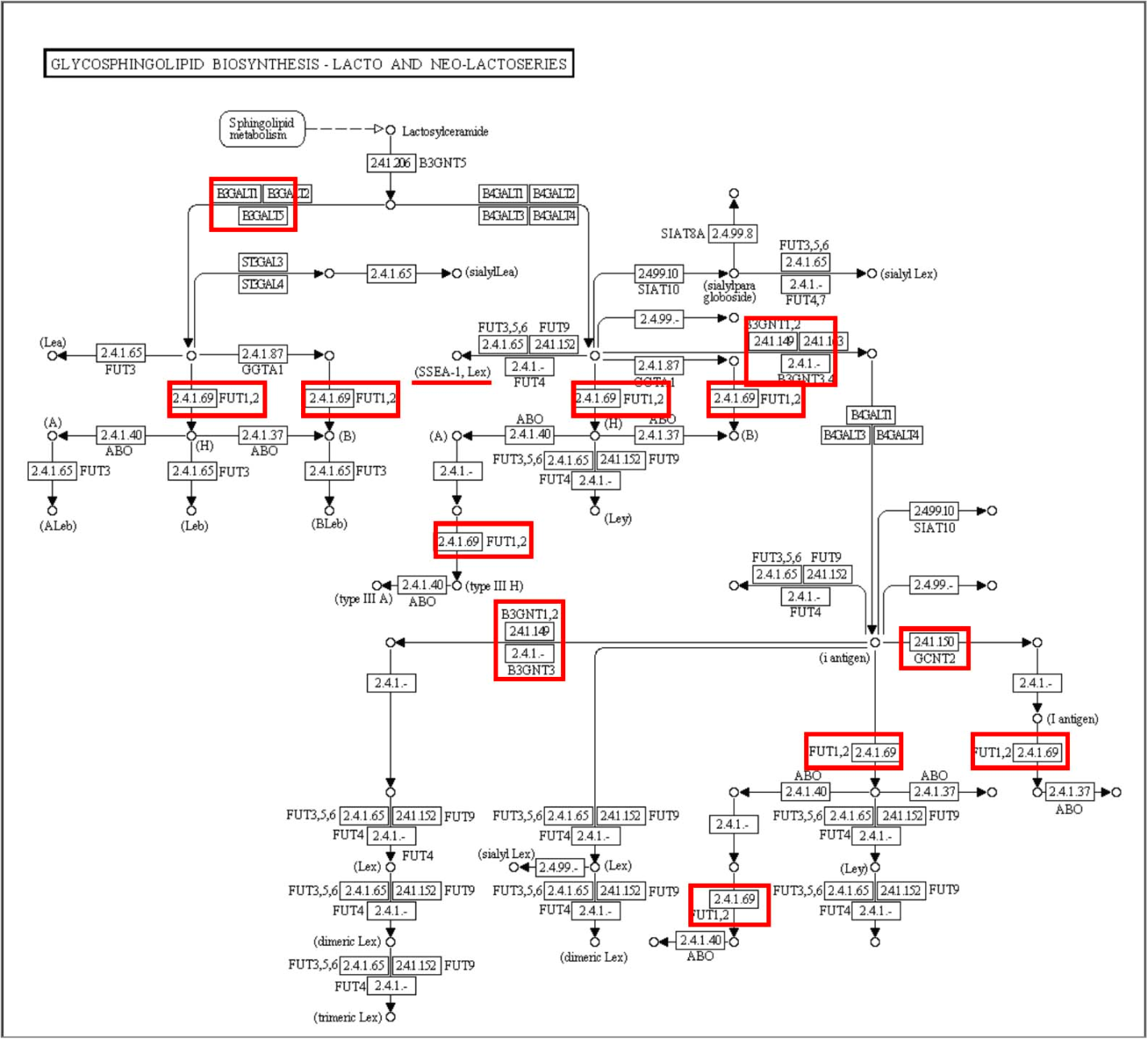
**

**
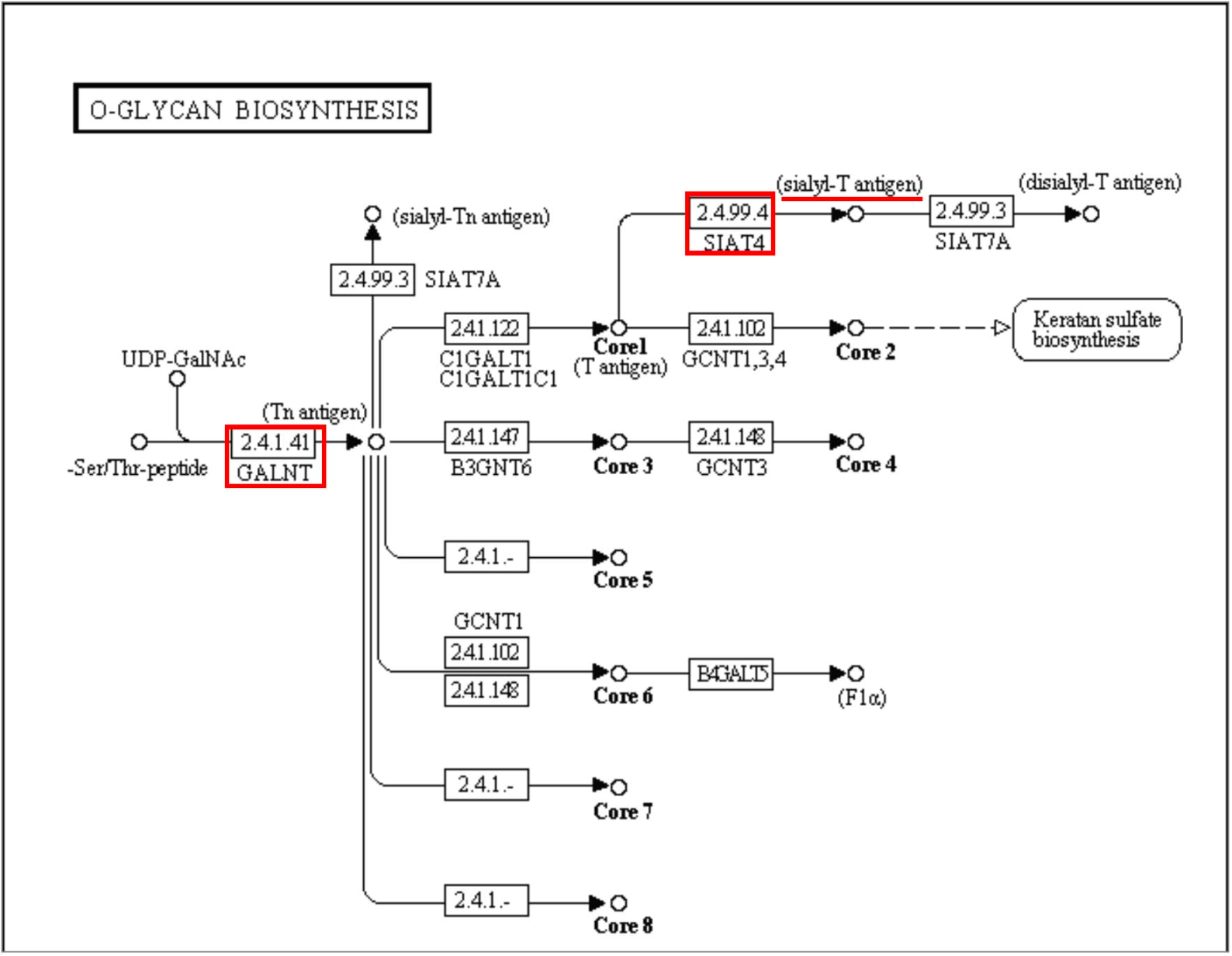
**

**
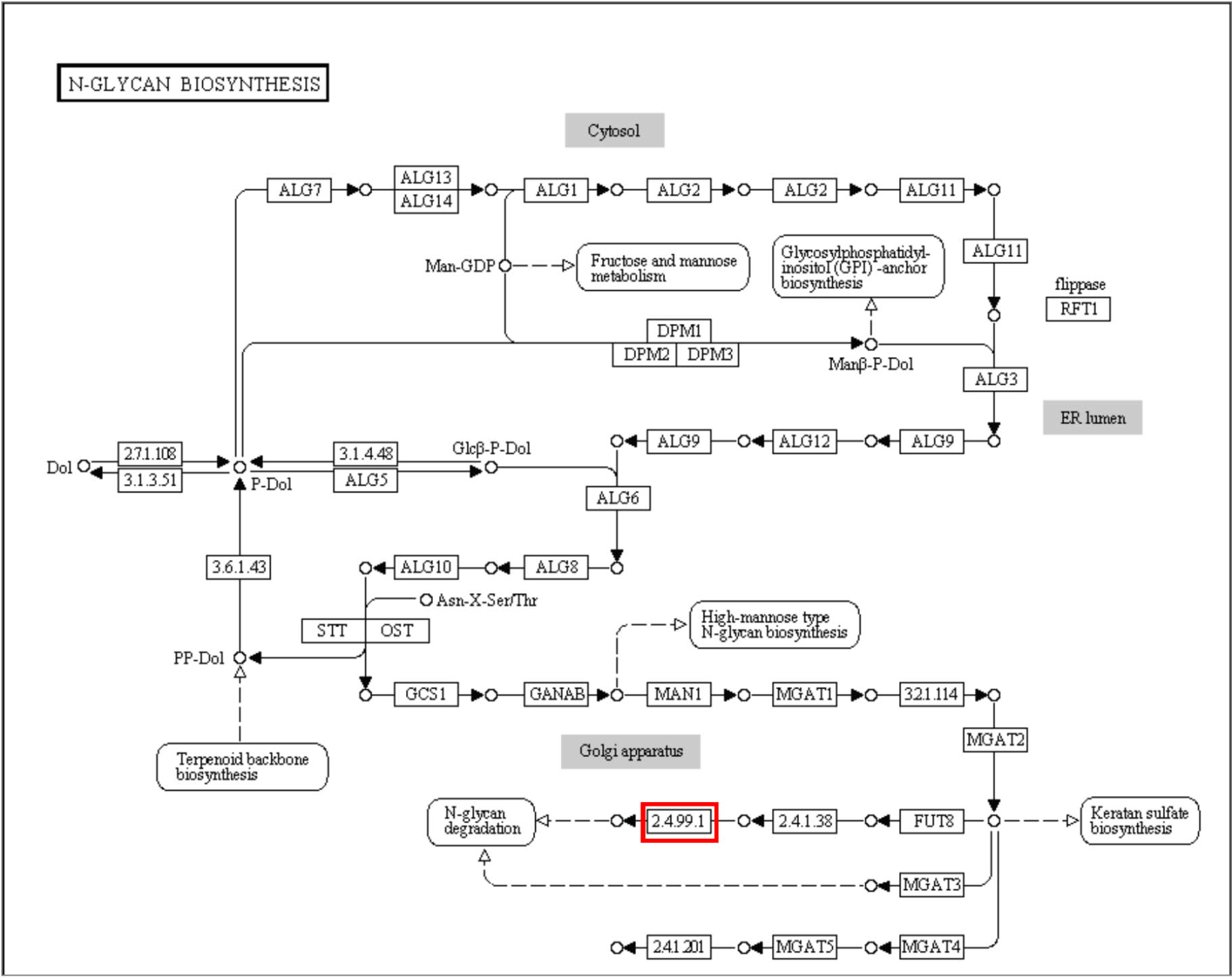
**

**Additional file 14: Locations of the glycosyltransferases detected in the present study in the pathways of “Glycan Biosynthesis and Metabolism”**

The glycosyltransferases listed in Table 1 were allocated to the pathways in “1.7 Glycan Biosynthesis and Metabolism” of the KEGG GLYCAN program (http://www.genome.jp/kegg/pathway.html#glycan). The glycosyltransferases and epitopes related to differentiation are indicated by red-colored boxes and red lines, respectively, in each pathway (see the text for details).
